# Wearable cardioverter defibrillator after ICD-system explantation: data from a multicenter registry

**DOI:** 10.1038/s41598-025-91046-4

**Published:** 2025-03-01

**Authors:** Ibrahim El-Battrawy, Thomas Beiert, Katharina Koepsel, Boldizsar Kovacs, Tobias C. Dreher, Christian Blockhaus, David Tenbrink, Norbert Klein, Thomas Kuntz, Hendrik Lapp, Dong-In Shin, Mohammad Abumayyaleh, Ardan Muammer Saguner, Mido Hijazi, Julia W. Erath, Firat Duru, Andreas Mügge, Ibrahim Akin, Assem Aweimer

**Affiliations:** 1 Department of Cardiology and Rhythmology, St. Josef Hospital, Ruhr University of Bochum, Bochum, Germany; 2 Department of Molecular and Experimental Cardiology, Institut Für Forschung Und Lehre (IFL), Ruhr University Bochum, Bochum, Germany; 3 Department of Internal Medicine II, University Hospital Bonn, University of Bonn, Bonn, Germany; 4 Department of Cardiology, University Heart Center, University Hospital Zurich, Zürich, Switzerland; 5 Department of Cardiology, Heart Centre Niederrhein, Helios Clinic Krefeld, Krefeld, Germany; 6 Faculty of Health, School of Medicine, University Witten/Herdecke, 58448 Witten, Germany; 7 Department of Cardiology, Angiology and Internal Intensive-Care Medicine, Klinikum St. Georg gGmbH Leipzig, Delitzscher Straße 141, 04129 Leipzig, Germany; 8 Department of Cardiology, Frankfurt University Hospital, Goethe University, Frankfurt am Main, Germany; 9 Department of Neurosurgery, University Hospital Carl Gustav Carus, Technische Universität Dresden, Fetscherstr 74, 01307 Dresden, Germany; 10 First Department of Medicine, University Medical Center Mannheim, Mannheim, Germany

**Keywords:** Cardiology, Medical research

## Abstract

Data on the use of the wearable cardioverter defibrillator (WCD) among patients after cardiac implantable electronic device explantation of 1- to 3-chamber implantable cardioverter defibrillator systems (ICD) are sparse. Accordingly, several guidelines give a different recommendation regarding WCD indication in this cohort. We aimed to study the baseline characteristics and outcome of patients treated with WCD after ICD explantation. The primary outcome is appropriate WCD shock. Within a multicenter registry 109 patients received a WCD to bridge the time after ICD-system explantation until reimplantation due to a persistent ICD-indication. The mean follow-up was 824 ± 773 days. In addition to ventricular tachyarrhythmias and/or WCD shocks during WCD wear time, also the rate of rehospitalization for ventricular tachyarrhythmias, atrial fibrillation, stroke and congestive heart failure after ICD-reimplantation was evaluated. Patients had a mean age of 65 ± 14 years, and were hospitalized for 21 ± 15 days. The index left ventricular ejection fraction (LVEF) was at baseline 35.7 ± 14.1% and 35.7 ± 14.2% at short-term follow-up. Mean wear time of the WCD was 61 ± 46 days after ICD-system explantation. During that time an appropriate WCD shock was documented in 7.3% of patients. Up to 80.6% of patients after ICD-system explantation were re-implanted. The rates of rehospitalization due to ventricular tachyarrhythmias, heart failure and atrial fibrillation were 7.3%, 6.8% and 4.1%, respectively. After ICD-reimplantation the rate of appropriate shocks was 12/89 (13.4%). Occurrence of malignant ventricular tachyarrythmia after ICD-system explantation is high and the use of WCD among these patients could be beneficial in preventing sudden cardiac death.

**Supplementary Information:**

The online version contains supplementary material available at 10.1038/s41598-025-91046-4.

## Introduction

A wearable cardioverter defibrillator (WCD) received a class II recommendation in varying guidelines e.g. American Society of Cardiology, European Society of Cardiology (ESC), German Society of Cardiology (DGK) and Austrian Society of Cardiology in different diseases to prevent sudden cardiac death (SCD)^1^. However, the strength of this recommendation is variable depending on the underlying cause for WCD prescription^2^.

Regarding WCD use after explantation of implantable cardioverter defibrillator (ICD-systems), there exist different recommendations (ESC guideline: IIb, Austrian guideline: IIa), whereas the German Society of Cardiology (DGK) does not publish any recommendations regarding this issue. This might be related to the sparse data from European countries. The American Heart Association and American College of Cardiology give a IIa recommendation for WCD use after ICD-system explantation.

Several guidelines suggest a prompt removal of the whole ICD-system and administration of intravenous antibiotics in case of infection^3–6^. This process may last from one week to six weeks or longer. Postponing ICD-system reimplantation would be helpful to complete the antibiotic treatment and to avoid reinfection. During the time of treatment and additional diagnostic procedures, patients may suffer from cardiac arrest related to ventricular tachyarrhythmias. Data from the United States and single center studies showed that the WCD may help to overcome this high risk in patients until reimplantation of an ICD^7–10^.

In the present paper we investigated 109 consecutive patients from seven hospitals in Germany and Switzerland receiving a WCD after ICD-system-explantation. To the best of our knowledge, only one report including 21 patients from European countries was published before^10^. The mean follow-up time in the present cohort is 824 ± 773 days.

## Methods

### Patient recruitment

Data of 109 patients with WCD prescription for ICD explantation due to an infection and/or lead dysfunction between April 2012 and March 2021 were extracted at seven university hospitals in Germany and Switzerland (University Medical Center Mannheim, Frankfurt University Hospital, Heart Center Leipzig, Bergmannsheil University medical center of the Ruhr-University, University Hospital Bonn, Helios Clinic Krefeld Germany, University Witten/Herdecke, and the University Hospital Zurich). A ZOLL Life Vest System was prescribed. The treatment regimen for infection varied and to protect the patient from ventricular arrhythmia during this time the WCD was used. The study was approved by the local ethics committee of University Medical Center Mannheim, Frankfurt University Hospital, Heart Center Leipzig, Bergmannsheil University medical center of the Ruhr-University, University Hospital Bonn, Helios Clinic Krefeld Germany, University Witten/Herdecke, and the University Hospital Zurich. Written informed consent was obtained from the participants. Patients confirmed participation. Informed consent has been obtained from all participants.

## The wearable cardioverter-defibrillator (WCD)

The registry, the programming of the WCD ZOLL Life Vest ™system (Pittsburgh, USA) and analysis of programmed data have been recently described^11–13^. As recommended the ventricular fibrillation (VF) zone was programmed at a heart rate of 200–220 bpm with a response time of 25 s. The maximum first shock energy was 150 J. The arrhythmic events were reviewed and classified by independent physicians. They were defined as sustained ventricular tachycardia (VT) (lasting 30 s or longer) or ventricular fibrillation (VF) with WCD shock therapy and non-sustained VT (lasting less than 30 s) without WCD shock. Inappropriate WCD therapy was identified as a non-ventricular tachyarrhythmia episode treated by an inappropriate WCD shock.

## Baseline and follow up data collection

The left ventricular ejection fraction was measured by the biplane Simpson’s method using echocardiography and/or cardiac magnetic resonance imaging (MRI). The ECG data (PQ, QRS and QT interval) and the New York Heart Association (NYHA) classification were evaluated at baseline, at three months (short-term) of follow-up and at six to twelve months (long-term). All data were retrospectively collected clinically and retrieved from the ZOLL Life Vest Network™. For follow-up data treating hospital archives were screened and physicians or patients were contacted. Different comorbidities (arterial hypertension, diabetes mellitus, stroke, atrial fibrillation and coronary artery disease) were extracted. Furthermore, drugs at discharge were documented. ICD-system implantation and follow-up data including cardiovascular hospitalization (stroke, congestive heart failure, atrial fibrillation, ventricular tachyarrhythmias, cardiovascular death) were additionally filled through a survey.

No standard protocol was prepared for prolongation of WCD use in recruiting centers.

Extraction of ICD-systems and re-implantation was done according to recent standard of care based on HRS and EHRA position papers.

## Results

### Description the cohort

In the present study we included 109 patients who received a WCD after ICD-system explantation, Table 1. The mean follow-up time was 824 ± 773 days. Patients had a mean age of 65 ± 14 years. The length of hospital stay was 21 ± 15 days. The index LVEF 35.7 ± 14.1%. Relevant comorbidities were chronic obstructive pulmonary disease (25.4%), atrial fibrillation/flutter (47.2%) and arterial hypertension ((59.7%). 28.8% of patients were smokers. At discharge patients received beta-blockers (83%), angiotensin-converting-enzyme inhibitors (ACEI) or angiotensin receptor blockers (ARBs) (49%), and aldosterone-antagonists (43%), Table S1.

**Table 1:** Baseline characteristics of the included patients.

Variables	Explantation (n=109)
**Demographics**	
Male. n (%)	90/109 (82.6%)
Age. mean±SD	65±14
Age ≥45. n (%)	98/109 (89.9%)
**Hospital side parameters**	
Cardiogenic shock at diagnosis. n (%)	11/59 (18.6%)
Pulmonary edema. n (%)	3/59 (5.1%)
Days of hospitalization. mean±SD	21±15
Clinic Treatment Results	
MRI	4/93 (4.3%)
Late gadolinium enhancement	3/93 (3.2%)
**LVEF and NYHA classification**	
Index LVEF	35.7±14.1%
Short-term LVEF	35.7±14.2%
Long-term LVEF	35.1±15.7%
EF Improvement >35%	40/76 (52.6%)
*NYHA index*	
1	16/78 (20.5%)
2	28/78 (35.9%)
3	31/78 (39.7%)
4	3/78 (3.8%)
*NYHA short-term*	
1	16/61 (26.2%)
2	24/61 (39.3%)
3	20/61 (32.8%)
4	1/61 (1.6%)
*NYHA long-term*	
1	2/10 (20%)
2	4/10 (40%)
3	4/10 (40%)
4	0/10 (0%)
BNP short-term. pg/ml (mean±SD)	1591.7±2075.1
BNP long-term. pg/ml (mean±SD)	969.4±586.1
**Comorbidities**	
CAD	30/59 (50.8%)
Myocardial infarction	22/67 (32.8%)
CABG	8/67 (11.9%)
COPD	15/59 (25.4%)
CKD/Dialysis	13/59 (22%)
Atrial fibrillation/Atrial flutter	50/106 (47.2%)
TIA/Stroke	6/59 (10.2%)
Diabetes mellitus	20/59 (33.9%)
Smoker	17/59 (28.8%)
Hypertension	40/67 (59.7%)
Hyperlipedemia	33/59 (55.9%)
Overweight	45/61 (73.8%)
Body mass index. mean±SD	28.7±5.9
Family history of cardiovascular disease	8/59 (13.6%)

### WCD data and follow-up of ICD-system

Regarding the explantation procedure in 41% of patients one lead, in 31.5% two leads, in 22% three leads, in 4.1% four leads and in 1.3% five leads were extracted.

**Fig. 1:**
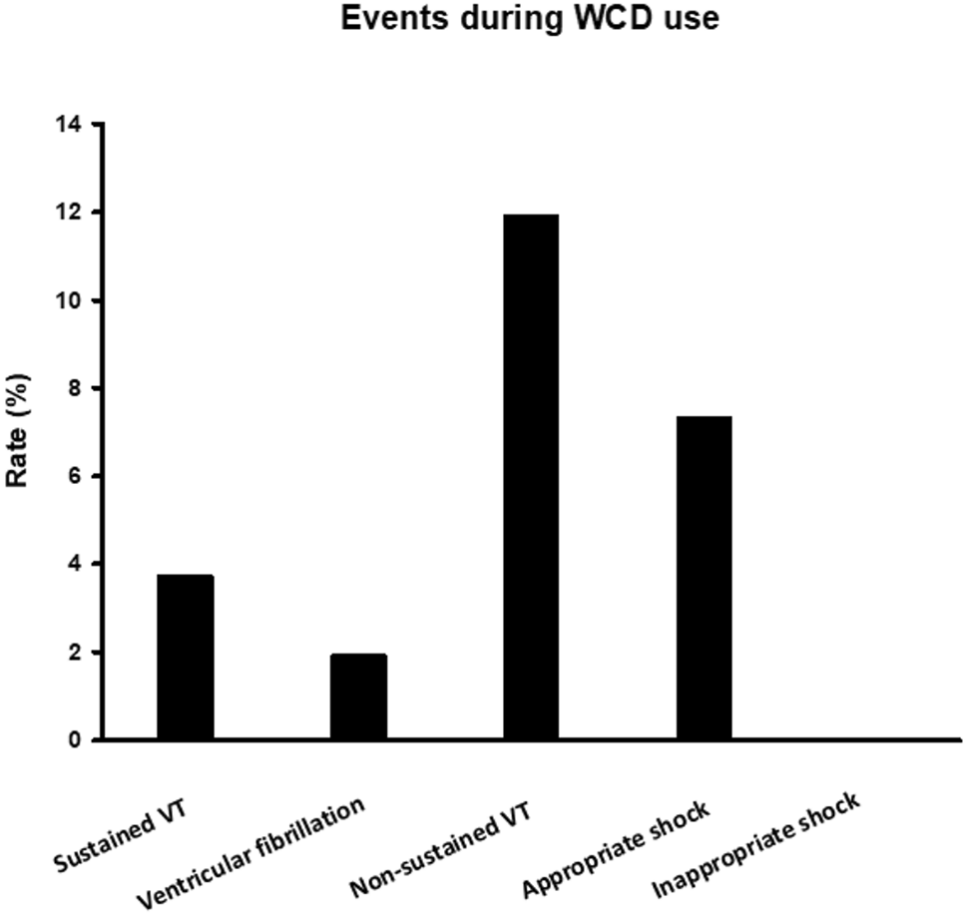
Rate of ventricular tachyarrhythmias, appropriate and inappropriate WCD shocks.

Wear days among patients after ICD-system explantation were 61 ± 46 day and only 17.4% of patients wearing the WCD for > 90 days, Table 2**.** Average wear time was 22.24 ± 4.04 h per day and patient compliance, defined as wear time > 20 h per day, was at 89.9%. The most common arrhythmic event was a non-sustained ventricular tachycardia with a rate of 11.9%. Sustained ventricular tachycardia and ventricular fibrillation were at 5.5% and 1.8%, respectively. An appropriate WCD shock was documented in 8 patients (7.3%) and the rate of inappropriate shocks was at 0%, Fig. 1. Among reasons for stopping WCD use, the ICD-system reimplantation was the most common with 80.6%. Other reasons for stopping WCD use e.g. incompliance, decision pending, no arrhythmic events, and death were at a low rate, Table 2.

**Table 2:** Data of patients with use of wearable cardioverter defibrillator due to ICD explantation.

Variables	Explant
	(*n* = 109)
Wear Days. mean ± SD	61 ± 46
Average wear time (hours). mean ± SD	22.24 ± 4.04
More than 90 Wear Days. n (%)	19/109 (17.4)
*Arrhythmic Episodes during WCD use*:	
Ventricular tachycardia. n (%)	6/109 (5.5)
Ventricular fibrillation. n (%)	2/109 (1.8)
Non-sustained ventricular tachycardia. n (%)	13/109 (11.9)
*WCD shocks*	
Appropriate. n (%)	8/109 (7.3)
Inappropriate. n (%)	0/109 (0)
**Reason for stopping WCD**	
Improved LVEF. n (%)	2/67 (3)
Implantation/planed implantation. n (%)	54/67 (80.6)
Incompliance. n (%)	0/67 (0)
Death. n (%)	2/67 (3)
No ventricular arrhythmias more post myocardial infarction. n (%)	2/67 (3)
Unknown. n (%)	6/67 (9)
Decision pending. n (%)	1/67 (1.1)
**Cardiac electronic device informations**	
Appropriate shocks (%)	12/89 (13.4)
*Arrhythmic episodes post-device implantation*	
Ventricular tachycardia (%)	5/67 (7.5)
Ventricular fibrillation (%)	1/67 (1.5)
Non-sustained ventricular tachycardia (%)	11/75 (14.7)
**Follow up Data**	
Follow up time	824 ± 773
-Death during follow-up (%)	15/109 (13.8)
*Rehospitalization (%)*	28/81 (34.6)
-Cardiovascular cause (%)	14/74 (18.9)
-Stroke (%)	0/74 (0)
-Ventricular tachycardia/ventricular fibrillation (%)	6/82 (7.3)
-Congestive heart failure (%)	5/74 (6.8)
-Atrial fibrillation (%)	3/74 (4.1)
-Any other cause (%)	7/72 (9.7)
**Patient-Compliance**	
Compliance (%)	98/109 (89.9%)

SD. Standard deviation; ECG. Electrocardiogram; EF. Ejection fraction; BMI. body-mass-index. LVEF. left ventricular ejection fraction. WCD; wearable cardioverter defibrillator. COPD; Chronic obstructive pulmonary disease; ACE; Angiotensin-convetring-enzyme. MRI; magnetic resonance imaging. NYHA; New York Heart Association. CAD; coronary artery disease. COPD; chronic obstructive pulmonary disease. CKD; chronic kidney disease

Post ICD-system implantation the rate of non-sustained ventricular tachycardia was the highest with 14.7%, followed by sustained ventricular tachycardia with 7.5%, and ventricular fibrillation 1.5%, Table 2.

Over a mean follow-up of 824 ± 773 days the death rate was at 13.8% and the rehospitalization rate was at 34.6%. The highest rate of rehospitalization was due to a cardiovascular cause (18.9%). Ventricular tachycardia or ventricular fibrillation was found as the reason for rehospitalization at a rate of 7.3% and congestive heart failure at a rate of 6.8%.

## Detailed investigation of patients with an appropriate WCD shock after ICD-system explantation

Eight patients after ICD-system explantation received an appropriate WCD shock. Among these patients only two suffered from ventricular tachyarrhythmias (former VF/VF) prior to ICD-explantation. Among the eight patients with appropriate WCD shock after ICD-system explantation one patient denied ICD reimplantation, one patient did not receive an ICD and another patient died for unknown cause, Table 3. The mean EF of these eight patients was 36.5%. Of note, the LVEF was ≥ 35% at time of initiating WCD use in four patients. These eight patients wore the WCD up to 166 days after ICD-system explantation. However, four patients used the WCD for only 40 days.

**Table 3:** Data of patients with use of wearable cardioverter defibrillator due to ICD explantation.

Gender	Age	LVEF base	Shock appropriate	Arrhythmic episode	ICD implant	Former VT/VF	Death during follow-up	Cause of death	Wear days
m	48	30	Yes	VT/VF	Yes	0	0	No	166
m	64	25	Yes	VT/VF	Yes	0	1	Unknown	40
m	71	27	Yes	VT/VF	Yes	0	0	No	18
m	64	40	Yes	VT/VF	Patient denied	Unknown	0	No	103
m	62	20	Yes	VT/VF	Yes	Unknown	0	No	104
m	64	40	Yes	VT	No	Unknown	0	No	1
m	67	35	Yes	VT/VF	Yes	1	0	No	5
f	44	75	Yes	VT/VF	Yes	1	0	No	117

## Discussion

We may conclude the following from the extracted sub-analysis from a multicenter WCD registry: (i) In patients with use of WCD due to ICD-system explantation the rate of appropriate WCD shock is high (7.3%); (ii) The average wear hours are high consistent with a high compliance rate; (iii) ICD-system explantation might be associated with increased ventricular tachyarrhythmia even if no prior event was observed by the ICD.

Several common clinical scenarios may lead to an ICD-system explantation e.g. ICD malfunction, lead fracture, device-and/or lead infection and in worst case scenario endocarditis^14^. Device-related infection and/or endocarditis require delayed reimplantation after ICD-system explantation to avoid reinfection. Within this time a prolonged antibiotic drug treatment and repetitive sampling of blood cultures is suggested. As soon as an ICD-system is reimplanted patients could be discharged. However, this process is associated with high costs, since the antibiotic therapy requires several weeks of in-hospital treatment with ECG-monitoring due to the lack of protection via an ICD. In addition, an earlier-implantation of ICD-system is not recommended to avoid a re-infection ICD-system. To minimize cost and to discharge patients early the use of a WCD may be favorable. The use of a WCD for this indication has been confirmed in multicenter and single center studies^12,13,15,16^. In a national registry published by Ellenbogen et al. in 8,058 patients, who received ICD-system explantation, 334 patients suffered from ventricular tachyarrhythmias. Consequently, the appropriate WCD shock rate per patient seems to be up to 4%^6^. Tanawuttiwat et al. reported in a single center study in the US that the SCD rate after ICD-system explantation is up to 4% in a cohort of 97 patients^8^. Kaspar et al. reported in a retrospective study data including 102 patients from a two centers that the rate of ventricular tachyarrhythmias was 8.8%. However, these data were related to a long-term use of WCD up to 638 ± 361 days^8^. To the best of our knowledge only one study in 21 patients after ICD-system explantation investigated the role of WCD in Europe. In this single center study one patient received an appropriate WCD shock consistent with a rate of 4.5%^10^. Our multicenter study including a sufficient number of patients from several European centers presents a sustained VT rate of 5.5%, VF rate of 1.9% and a non-sustained VT rate of 11.9% recorded by the WCD. This rate seems to be comparable to previous reports. In addition, we show an appropriate WCD shock rate of 7.3%, which is the highest reported among this patient group. Of note, our data show that the use of a WCD in patients with ICD-system explantation is safe. For example, we present an inappropriate WCD shock rate of 0%. Other data from the reported studies on WCD use among patients after ICD-system explantation show an inappropriate WCD shock rate of up to 5.8%. This low rate of inappropriate WCD shocks among this cohort may be related to two factors. First, the mean wear days (61 ± 46 days) was shorter compared to other published data and second there might be differences in training and instruction of patients when receiving a WCD. Of note, data have shown that the WCD use was less expensive compared to standard therapy (low-intensity inpatient hospitalization). The cost-minimization analysis showed a cost reduction of 1782€ per patient using the WCD^17^. Taking together the favorable clinical outcome of patients treated with a WCD and the beneficial economic aspects of WCD use after ICD-system-explantation, this might strengthen the role of the WCD for this specific indication. Current guidelines classify the WCD after ICD-system explantation as follows: Class IIa/B in the American guidelines^18^, IIb/C in the European Society of Cardiology guidelines^19^and IIa/C in the Austrian guidelines. The German guidelines give no specific recommendation^1^.

But nevertheless, when European and German guidelines are dissected regarding this classification, it seems that the weakness of recommending WCD in patients after ICD-system-explantation could be based on missing data from European countries at time of guideline development. This issue should be reevaluated in future guidelines and position papers.

In addition, we show in the present analysis that among patients after ICD-system explantation the rate of rehospitalization due to congestive heart failure is 6.8% which might be related to the role of reduction and/or absence of biventricular stimulation due to lead extraction or due to additional extraction of CCM leads in the same procedure. Among the whole WCD cohort in 41% one lead, in 31.5% two leads, 22% three leads, 4.1% four leads and 1.3% five leads were extracted. Another aspect could be the need for hydration due to infection and/or sepsis which may affect rehospitalization of patients due to congestive heart failure.

Published data on WCD use after ICD-system explantation showed that the occurrence of ventricular tachyarrhythmias was noted in the initial weeks after ICD explantation. This rate was 0.9% in the first week and decreased to 0.7% in the second and third week, resulting in a cumulative event rate at the end of 1 year of 10%^6^. Of note, we show a high rate of ventricular tachyarrhythmias within the use of WCD over 61 ± 46 days, which might even be higher if the wear time was prolonged to 365 days (1 year). This high initial rate of ventricular tachyarrhythmias illustrates the need for anti-arrhythmic protection and may be triggered by several factors. Infection and/or endocarditis are associated with cardiac emboli, which may exacerbate ventricular tachyarrhythmias^20,21^. Further important triggers might be the extraction of leads itself. Of note, among patients after ICD-system explantation the reimplantation rate was up to 80.6%. This rate is comparable to published data.

## Conclusion

WCD use after ICD-system explantation seems useful due to a high rate of ventricular tachyarrhythmias. The rate of appropriate WCD shock was 7.3% whereas no inappropriate WCD shocks were documented. ICD-system explantation might be associated with an increased risk of ventricular tachyarrhythmias.

### Study limitation

The main limitation of this study is the retrospective nature of data collection and analysis. Furthermore, heterogeneity of data and bias are not excluded. The study did not compare patients treated with a WCD after ICD-explantation to those without WCD. In addition, data about length of ICD therapy before explantation, the time of intervention and the site (in or out of hospital) were not available. Future randomized trials are required to confirm the present results.

## Electronic supplementary material

Below is the link to the electronic supplementary material.

Supplementary Material 1

## Data Availability

The datasets used and/or analysed during the current study available from the corresponding author on reasonable request.
