# Supplementary material for: Wearable cardioverter defibrillator after ICD-system explantation: data from a multicenter registry
Source: Sci Rep. 2025 Mar 1;15:7270. doi: 10.1038/s41598-025-91046-4 (PMC11873030; doi:10.1038/s41598-025-91046-4)
Supplement: Supplementary file 1 — Supplementary Material 1 [file 41598_2025_91046_MOESM1_ESM.docx]

| **Table S1: drug history at discharge of the included patients** | |
| --- | --- |
| **Variables** | **Explantation**  **(n=109)** |
| Statine | 43/67 (64.2%) |
| ACE inhibitors a/o ARBs | 33/67 (49.3%) |
| ARNI | 7/67 (10.4%) |
| Aldosterone antagonist | 29/67 (43.3%) |
| Beta blockers | 85/102 (83.3%) |
| Any antiarrhythmic drug | 10/67 (14.9%) |
| Amiodarone | 16/107 (15%) |
| Diuretics | 50/67 (74.6%) |
| Digitalis | 4/67 (6%) |
| Procoralan | 8/67 (11.9%) |
